# Who Received Informal Social Support During the First COVID-19 Lockdown in Germany, and Who Did Not? The Role of Social Networks, Life Course and Pandemic-Specific Risks

**DOI:** 10.1007/s11205-022-02890-0

**Published:** 2022-03-14

**Authors:** Ariane Bertogg, Sebastian Koos

**Affiliations:** 1 Institute of Advanced Studies, Department of History and Sociology, University of Konstanz, Konstanz, Germany; 2 Cluster of Excellence “The Politics of Inequality”, Department of History and Sociology, University of Konstanz, Konstanz, Germany

**Keywords:** Social support, Informal Help, Need, Receiving, Solidarity, COVID-19 pandemic, Life Course, Social Networks

## Abstract

In this article, we study the receipt of informal support during the first wave of the COVID-19 pandemic in Germany. The containment measures have had various, far-reaching consequences for the wellbeing of people, creating demands for economic, practical, and emotional support—even among individuals who hitherto were not in need of support. Existing research has shown substantial levels of informal support during the pandemic, often based on individuals’ existing social networks, but has predominantly taken the perspective of donors. In this article, we focus on the “demand” or recipient “side” of informal support, and ask: (1) Who receives which type of informal social support during the pandemic? (2) Who reports unmet need? (3) Which factors explain support receipt, unmet need and the type of support received? To explain patterns of receiving social support, we identify “classic” life course and “new” pandemic-specific risks and complement this perspective with individuals’ support potentials from their social networks. Empirically, we use data from an online survey, collected among a quota sample of the German population (n = 4,496) at the end of the first lockdown in late spring 2020. Our analysis shows that one in six respondents received social support, while only 3% report unmet need. Practical and emotional support are most widespread. Using logistic and multinomial logistic regression models our results show that social support in general and the type of support received can be explained by life course and pandemic risks, while unmet need is mainly a consequence of social network structure.

## Introduction

Societal crises and natural disasters have always had dire social consequences, sometimes leaving large parts of a population in substantial need of social support (Kutak, 1938; Kaniasty et al., 1990; Kasapoglu et al., 2004). The COVID-19 pandemic and ensuing governmental lockdown measures have created existential challenges for the wellbeing of people across the globe, representing an almost universal crisis. In Germany, as of mid-March 2020, far-reaching lockdown measures were implemented in order to slow down the spread of the SARS-CoV2 virus. These measures resulted in a full halt of public life, creating demands for social support while at the same time strongly limiting opportunities for social contacts and exchange.

The containment interventions have on the one hand intensified pre-existing needs for support and, on the other hand, have created “new” needs among individuals who were previously not depending on external support. Indeed, first studies on the COVID-19 pandemic indicate that not receiving support despite being in need (unmet need) might indeed disproportionally affect those who were already in disadvantaged positions before the outbreak of the pandemic (Perry et al., 2021; Gauthier et al., 2021). With regard to pre-existing needs, people with medical pre-conditions or chronic diseases have experienced a shortage in routine healthcare treatments or medicament supply, due to the overburdened health system. Moreover, together with the elderly, they were defined as “risk groups” for severe COVID-19 pathologies (Jordan et al., 2020), and were strongly discouraged to leave their homes (Kushtanina & Vinel, 2020). From this *new, pandemic-specific risk*, a sudden demand for support with running basic errands has emerged among individuals from this group (Armitage & Nellums, 2020). In addition, diverse new needs arose, for instance due to the closing of schools and childcare facilities, the layoffs or working time reduction of many workers, and the uncertainty and psychological stress of the lockdowns (Bundesagentur für Arbeit, 2020; Kulic et al., 2020; Ohlbrecht & Jellen, 2020; Luiggi-Hernández & Rivera-Amador, 2020). These consequences have triggered heightened demand for childcare, private financial transfers, and emotional support.

Understanding when and how civil society ensures the well-being of people in need during a crisis, but also the limits of such support, is important, not only from a policy but also from an academic perspective. While the individual and contextual level determinants of receiving different forms of support (time, money, and emotional support) under “normal” circumstances have been widely studied (Brandt et al., 2009; Havens et al., 2006; Suanet & Antonucci, 2017; Künemund & Rein, 1999), fewer studies have addressed the impact of disasters and crises on receiving different types of support (Kasapoglu et al., 2004; Beggs et al., 1996; Kaniasty et al., 1990). Therefore, our study investigates the receipt of different types of social support during the first lockdown in spring 2020 in Germany.

We ask: (1) Who receives (different types of) social support during the COVID-19 crisis? (2) Who has unmet need? (3) Which factors explain who receives support and who does not? To explain patterns of receiving and not receiving social support we extend a classic model used in health research (Andersen & Newman, 1973), which we enrich with a life course and a social network perspective. The life course perspective allows to identify vulnerable groups with existing support needs (Taylor-Gooby, 2004), which may have intensified due to the pandemic (Holst et al., 2020; Fortier, 2020; Douglas et al., 2020). Support receipt, however, is not only based on need alone (Künemund & Rein, 1999). Thus, the social network perspective allows to identify the available support potentials and explains why some individuals have not received support despite being in need (Brown & Ferris, 2007; Havens et al., 2006). Third, we introduce the concept of new, pandemic-specific risks. These represent the specific ways in which the lockdown measures have affected individuals, previously independent of support, particularly those who belong to a risk group.

We base our empirical analyses on an online survey, which was conducted at the end of the first lockdown in Germany, when most far-reaching lockdown measures were still in force. Based on a quota sample of the adult population, 4,496 respondents were surveyed about their perceptions of and behaviour during the COVID-19 crisis, with a focus on social inequality, pandemic governance, and solidarity. To answer our research questions, we use detailed measures of different types of informal social support and unmet need. We analyse three dependent variables: the receipt of social support, the type of support, and whether one has unmet need.

Germany is a well-suited context to study informal support during the pandemic. The initial lockdown measures were less strict than in some other countries, such as Italy or Spain, which were hit harder by the pandemic. However, they were comparable to the first response of many other countries in Europe, such as Austria or Denmark (Hale et al., 2021). The corporatist German welfare regime (Esping-Andersen, 1990) provides considerable formal support for people in need due to “classic” social risks (Taylor-Gooby, 2004). Yet, similar to other European countries, such as Denmark, the first lockdown increased the demand for informal social support, especially for newly dependent individuals, due to pandemic-specific risks (Andersen et al., 2020). Thus, the situation of German citizens in need of support during the first wave of the pandemic is likely to be comparable to the situation in other continental European countries.

The remainder of this article is structured as follows: In the next section, we discuss the theoretical background. The analytical strategy is in the subsequent section, together with a description of our data, variables, and method. The fourth section presents the results. It is followed by a discussion and conclusionL section, in which we summarize and discuss our findings, discuss the limitations, highlight the policy implications of this study, and avenues for future research.

## Theoretical Background and Previous Research

The individual and contextual-level determinants of receiving time, money, and—to a lesser degree—emotional support, under “normal” circumstances have been widely studied (see, e.g., Brandt et al., 2009; Suanet & Antonucci, 2017). These studies suggest that needs which arise during the life course (e.g., due to age, health problems and frailty, but also at the transition to parenthood or when becoming unemployed), are decisive to explain the receipt of support (Broese van Groenou & De Boer, 2016). Moreover, they indicate family members play an important role in providing social support. Non-family ties, such as friends and neighbours mainly step in when partners and children are not around, or when they are living too far away (Schnettler & Wöhler, 2015).

Fewer studies have addressed the impact of disasters and crises on receiving different types of informal social support (Kasapoglu et al., 2004; Beggs et al., 1996; Kaniasty et al., 1990). In addition, so far we know little about who does not receive support despite being in need during a crisis (Perry et al., 2021; Gauthier et al., 2021). By “social support” we hereafter refer to any type of informal help being received privately from others, be it time, money or goods, or emotional counselling and comfort. We exclude formal services provided by governments (e.g., meals on wheels), or that are privately paid for (e.g., a cleaning help). We thus rely on a relatively broad conceptualization which is widely established in the literature (for a summary of definitions, see Barrera & Ainlay, 1983).

### An Explanatory Model for Receiving Support

We argue that the receipt of (different types of) support during the COVID-19 pandemic support follows a complex pattern. This was already proposed in an early model by Andersen and Newman (1973), which was developed to predict healthcare uptake^1^. The model identifies three groups of factors, which lead to individuals seeking support, namely: need (such as life course or other risk factors), enabling (such as network embeddedness) and predisposing factors (such as socio-demographic factors, available formal services, but also attitudes). These factors may entail the characteristics of individuals (e.g., their frailty status), their networks (e.g., the availability of helpers), or the community they live in (e.g., the formal care infrastructure). However, there is little theoretical reasoning about the mechanisms behind the specific indicators utilized to depict these three groups of factors. Moreover, this theory was developed to explain healthcare usage rather than informal support, due to acute need. Hence, more specific theoretical mechanisms are needed to identify the relevant determinants of needing and receiving various types of support, and these mechanisms have to be linked to the specific situation of the pandemic.

**Fig. 1:**
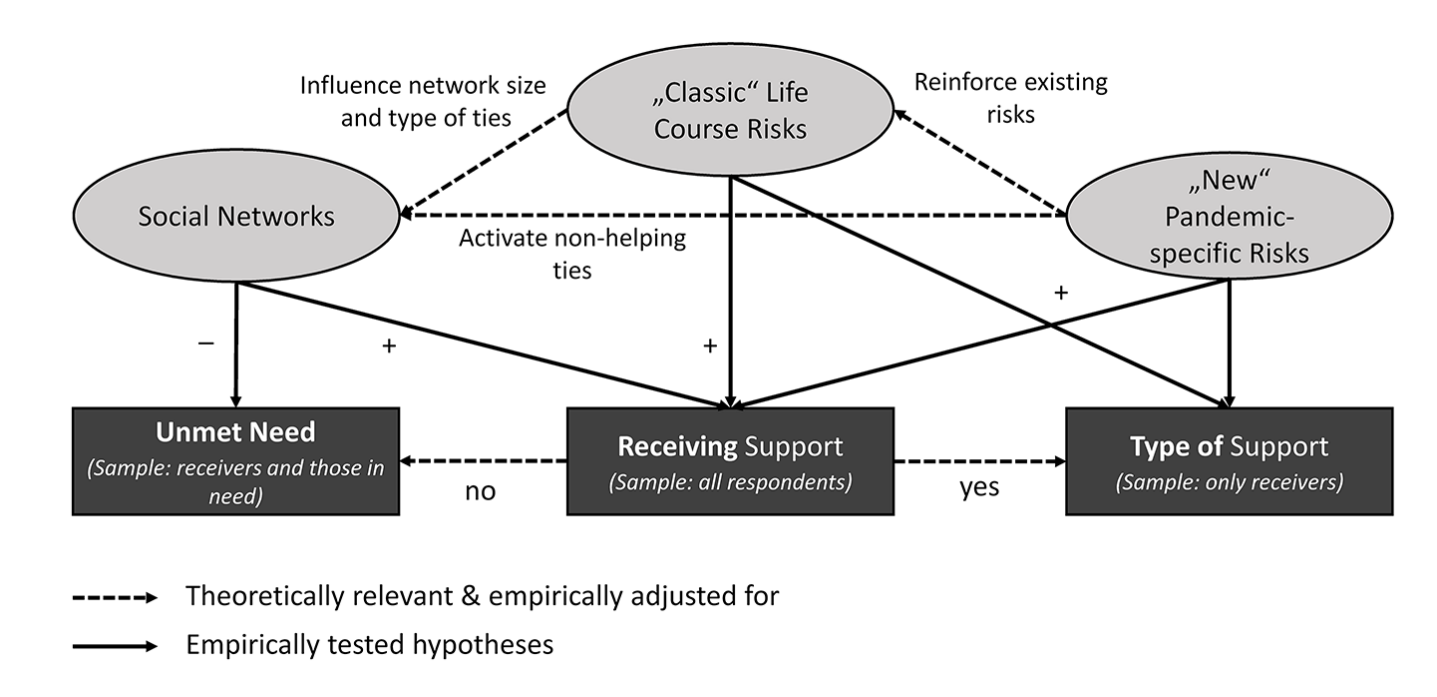
Theoretical model. Legend: Own illustration (analysed samples in parentheses)

To identify such mechanism, we enrich this model with a life course perspective and social network theory and add the concept of *pandemic-specific support risks* in order to account for the extraordinary situation during the lockdown (see Fig. 1). We argue that both life course and pandemic-specific risks allow a more nuanced understanding of the specific situations that require support than in the classic model (Andersen & Newman, 1973). In addition, we discuss the importance of different social ties for receiving social support (Granovetter, 1983). Moreover, we discuss the implications of the pandemic for these mechanisms. Such a model allows us to explain who does and who does not receive support during the pandemic. Moreover, this model also helps to explain which type of support is needed, such as practical support, childcare, financial aid, or emotional support, as these arise from different needs.

*“Classic” life course risks* entail the risk potentials due specific stages in the life course and the transitions and situations typically accompanied therewith, such as being elderly, suffering from physical constraints, having small children, or not being employed, which explain why individuals need specific types of support (Taylor-Gooby, 2004). They are represented by the downward pointing, solid, arrows from “classic” life course risks to “Receiving” and “Type of Support”. ”*New, pandemic-specific risks”* arose during the pandemic (Settersten et al., 2020), most notably, an entire population group being defined as a “risk group”. They create specific support needs, which bypass “classic” life course risks, as represented by the direct downward pointing arrows. They may also reinforce existing life course risks, for instance putting already precarious workers in even more vulnerable positions (as depicted by the dashed arrow from “new” to “classic” risks).

The receipt of support depends not only on need alone (Künemund & Rein, 1999), but also on the availability of supporters in one’s *social network* (Antonucci et al., 2010). This is depicted by the direct arrow from “Networks” to “Receiving”. Networks are the channels though which demand for support is voiced and willingness to help is communicated (Brown & Ferris, 2007; Varese & Yaish, 2000). The structure of the social network—for instance distinguishing between strong and weak ties (Granovetter, 1983)—is particularly crucial in explaining why some individuals in need do not receive support (“Unmet Need”).

Networks are not independent of life course and pandemic-specific risks (the dashed lines). Size and structure of individuals’ network vary across the life course, and close ties influence each other’s needs and resources (“linked lives”, Landes & Settersten, 2019). Moreover, the lockdown measures may limit the support potentials of strong ties or activate ties other than the “traditional” support ties (Bertogg & Koos, 2021; Carlsen et al., 2020). We discuss these implications in more detail in the following sections.

### “Old” and “New” Risks for Support: Life Course and Pandemic-Specific Need

The life course framework (Elder, 1998; Mayer, 2009) provides an opportunity to explain why and when different types of *need* arise. As a multi-perspective, multi-dimensional framework, it allows us to integrate both support needs and support potentials in a person’s social network via the idea of “linked lives” (Landes & Settersten, 2019). The life course lens is thus very well suited to understand the complex patterns of need for and receipt of (different types of) social support during the pandemic (Settersten et al., 2020).

Broadly, four different stages in the adult life course can be distinguished: young adulthood, mid-adulthood, “young” old age, and “old” old age. These stages are typically characterised by specific life course transitions (e.g., from school to work, into or out of a partnership, into retirement, parenthood, widowhood, or frailty), and are linked to probabilities of having a certain health, family, and employment status, which links them to specific support needs (such as childcare support, personal care, practical help). Particularly, transitions between statuses constitute risks (Taylor-Gooby, 2004) and call for material, practical, and socio-emotional support to adjust to the changes they entail (Kafetsios, 2006; Thoits, 2011). Thus, different stages in the life course are associated with a different prevalence of the various types of support (Attias-Donfut et al., 2005; Broese van Groenou & De Boer, 2016).

In young adulthood, the transition into the labour market, partnership, and parenthood are pending or ongoing, increasing primarily the need for financial and childcare support (Arnett, 2000). Indeed, financial aid is most likely to be provided from older to younger generations (Attias-Donfut et al. 2005), and in most Western countries, young parents rely on informal support with childcare (Leopold & Skopek, 2015). In mid-adulthood and early old age, the density of life course transitions decreases, and individuals are more likely to be the givers than the receiver of support (Patterson & Margolis, 2019). In late old age, the number and density of life course transitions increases again, and so does the likelihood of receiving support (Kahn & Antonucci, 1980; Brandt et al., 2009). In old age, health typically declines, and chronic illnesses, mobility limitations and disabilities become more likely, limiting individuals’ abilities to take care of their household, preparing food, or getting dressed. In order to remain living in their homes, many old receive informal support (Brandt et al., 2009; Barnett et al., 2012). This age group is particularly likely to receive practical support, e.g., with running basic errands or help in the household (Suanet & Antonucci, 2017; Messeri et al., 1993). Thus, not only the likelihood, but also the types of support received vary between the different life course stages and can be explained by the health risks and parenthood status.

Besides these “classic” life course factors, the COVID-19 pandemic has made a new group of individuals dependent on social support. In Germany, a so-called “risk group” was identified on the basis of their increased risk of mortality or severe pathologies from COVID-19 (Jordan et al., 2020). It was defined by age (65 or older, despite being healthy otherwise) and chronic illnesses (such as diabetes, or immunity deficiency, which also applies to younger individuals). Individuals belonging to this group were asked to self-isolate in order to protect themselves during the first lockdown in spring 2020. Not being able to leave the house, members of this group became dependent on others, particularly to run errands for them.

### The Role of Social Networks for Receiving Support

According to the convoy model (Kahn & Antonucci, 1980), the social network of a person can be described with regard to its structure (the number of overall ties, the nature of these ties, the geographic proximity to the network members) and function (different types of support given and received). Social networks have been shown to be of crucial importance for receiving social support (Antonucci et al., 2010; Messeri et al., 1993; Schnettler & Wöhler, 2015). Yet, both the structure and composition of a person’s network vary over the life course. As people age social networks tend to become smaller, more informal, and kinship centred (Carstensen, 1992; Suanet & Antonucci, 2017). Despite shrinking networks, critical life events in old age, such as frailty or widowhood, activate social support (Broese van Groenou & De Boer, 2016; Riley & Riley, 1993).

A second perspective on social networks entails the distinction between “strong” and “weak ties” (Granovetter, 1983). ”Strong” respectively “weak” ties are often defined along the lines of kinship respectively level of formality (e.g., formal membership), but these dimensions overlap (Plickert et al., 2007; Putnam, 2000). Strong ties can entail both kinship (e.g., relatives) and non-kinship (e.g., close friends) ties, but are usually informal in nature. Weak ties can entail both informal (e.g., neighbours) and formal (e.g., colleagues, members of one’s association or congregation) network contacts, and are usually not kinship-based. Weak and strong ties have been associated with different types of support: while strong ties provide often emotional and practical support (Ermer & Proulx, 2019; Schnettler & Wöhler, 2015), or financial aid (Attias-Donfut et al., 2005), weak ties are more likely provide information, job opportunities and bridge otherwise unconnected networks (Granovetter, 1983). In the following, we detail the support potentials of the different strong and weak ties for different types of support and discuss the implications of the COVID-19 pandemic for the support potentials from these various ties.

With regard to strong ties, previous research has shown that kinship supporters are the preferred for personal care and health-related support needs (Messeri et al., 1993), reflecting normative expectations towards family members to support each other (Cooney & Dykstra, 2011). Family members also frequently support each other with practical support, such as housework or running errands (Brandt et al., 2009) as well as financially (Attias-Donfut et al., 2005). Under conditions of a lockdown, needs are more difficult to communicate. Therefore, we argue that the more frequent contacts were with family members before the pandemic—a proxy for quality of these ties—the more likely it should be that these support potentials are also used during the lockdown.

Partners and spouses occupy a special support role. They are the most likely source for health-related care, but also emotional support (Bertogg & Strauss, 2020; Ermer & Proulx, 2019). Living in a partnership entails further benefits such as pooling economic resources (Vandecasteele, 2010) or sharing domestic work (Grunow, 2019). Because partnership relations were least affected by the lockdown measures, living in a partnership should equip individuals with a broad range of support resources, This should make them less dependent on (external) support and decrease their risk for unmet need—even under conditions of a lockdown.

Despite this prime function of family ties in providing support, non-kin strong ties are important for practical support, too, for instance when a person does not have many family ties, such as partners and children (Sarkisian & Gerstel, 2015), or when family members are living too far away to provide support (Conkova et al., 2017; Messeri et al., 1993). The extraordinary situation of the pandemic, which involves health risks and travel restrictions, may indeed have made local, non-kin, ties a viable alternatives for otherwise lacking kinship support. Thus, frequent contacts with friends may also activate support during the pandemic and protect against unmet need.

With regard to weak ties, we distinguish between formal social capital, such as membership in an association or a religious community (Putnam, 2000), and informal ties, such as neighbours and colleagues with whom one has also private contacts. Most associations in Germany rely on unpaid volunteer work (Erlinghagen, 2010). Studies indicate that such formal volunteers are often intrinsically motivated and more likely to support others informally (Choi et al., 2007). Similarly, membership in a religious community increases the support potential from weak ties. Religious individuals, too, are more likely to volunteer or support others (Bekkers & Wiepking, 2011; Krause, 2015). Thus, both being a member of a formal association or regularly joining a religious community should increase the availability of support during the pandemic, because they increase the pool of motivated informal supporters.

Strong and weak ties were differently affected by the COVID-19 containment measures. Many individuals narrowed their in-person contacts to a core network of strong ties (Arpino et al., 2020), leaving these ties less affected by the contact restrictions. Meeting with weak ties, however, was more strongly discouraged, e.g., through mandatory working from home, and the closure of voluntary associations and churches. One could thus assume that formal weak support potentials were less accessible. In the context of the COVID-19 pandemic, however, emerging support arrangements were found to be based on both weak and strong ties (Carlsen et al., 2020; Gauthier et al., 2021). Particularly neighbourhood or online-based support networks could be observed during the pandemic (Carlsen et al., 2020; Bertogg & Koos, 2021). This might counterbalance the lost opportunities of interacting with formal weak ties. Thus, both strong and weak ties should matter for support receipt.

### Hypotheses

Our theoretical considerations can be summarized in a number of hypotheses. With regard to “classic” life course risks, we assume the following:

H1a: The youngest and the oldest age group are more likely to receive support than individuals in middle and early old age.

H1b: Parents of minor children are more likely to receive support, than childless respondents or respondents with adult children.

H1c: The more severe an individual’s health condition, the more likely they are to receive support.

With regard to the type of support received, we assume the following:

H2a: Respondents who belong to the youngest age group, as well as parents of minor children, are more likely to receive childcare or financial aid than the other age groups.

H2b: Respondents who belong to the oldest age group are more likely to receive practical support than those in other age groups.

H2c: The more severe an individual’s health issues the more likely they are to receive practical support.

Turning to the new, pandemic-specific risks, we expect the following:

H3a: Members of COVID-19 risk groups are more likely to receive support.

H3b: Members of COVID-19 risk groups are more likely to receive practical support.

As regard social networks, we distinguish between strong and weak ties. For strong ties we expect the following:

H4a: Partnered individuals are less likely to report receiving support than partnerless individuals.

H4b: Partnered individuals are less likely to report unmet need.

H4c: Respondents who met family members and friends, and colleagues more frequently before the pandemic are more likely to receive support during the pandemic.

H4d: Respondents who met family members, friends, and colleagues more frequently before the pandemic are less likely to report unmet need.

With regard to weak ties, we assume that:

H5a: Individuals who are a member of one or several voluntary associations or take part in religious meetings more frequently are more likely to receive support.

H5b: Individuals who are a member of one or several voluntary associations or take part in religious meetings more frequently have a lower likelihood of experiencing unmet need.

## Data and Methods

### Data

To test our hypotheses, we use of data from an online-survey, collected during the first COVID-19 lockdown in Germany (blinded). The survey was implemented in an online access panel which draws on a quota sample of the German population. Quotas were used for age, gender, education, and region, and were designed to approximate the distribution of these characteristics in the German population (using census data). A comparison of the resulting sample to German census data shows that our sample is very close to the general population in terms of these key sociodemographic characteristics (see table A.1 in the Appendix). The survey was fielded between 29th of April and 8th of May 2020, when far-reaching lockdown measures were still in action. It focuses on the impact of the COVID-19 pandemic on private lives and society. One of the survey modules asked for giving and receiving of different types of social support during the pandemic^2^.

During the first lockdown in spring 2020, it was highly difficult to collect data in any other way than online, even telephone surveys were difficult to administer, because the lockdown measures also affected survey providers and call-centres. Yet, we consider the timing of the survey highly relevant to understand the immediate impact of the (first) lockdown on local solidarity. The obvious downside is the potential digital divide and the underrepresentation of individuals without internet connection. Yet according to Eurostat, in 2020, 93% of Germans used the internet regularly (at least once a week) and 96% of all households had internet access (Eurostat, 2021). Despite this limitation, our data should thus be well suited to provide a good picture of the need and receipt of support during the first lockdown in the COVID-19 pandemic.

The data provider used several quality checks to identify and exclude speeders and straight-liners resulting in a sample of 4,799 respondents. For the analysis we excluded all respondents without valid observations on the dependent and independent variables (listwise deletion). Our final sample consists of 4,496 respondents. In the following, descriptive results are presented using weights, whereas multivariate models are estimated controlling for the respective variables that were used for the weighting. Additional models using weights (not reported) showed that results were highly consistent.

### Dependent Variables

We use *three dependent variables*. The first and second dependent variables are based on the following question: “In the last weeks since mid-March, the Corona-crisis and the measures associated therewith, such as curfews and closures of childcare facilities, have led to a situation in which many people now are in need of private support from others, for instance through shopping, childcare, emotional support or in other ways. During this time, did you:”, followed by five answering options^3^ “Offer help to someone (also via a platform)”, “Help someone”, “Need help”, “Ask someone for help (also via a platform)” and “Receive help”. Individuals could select all answering options that applied.

*Receiving* social support was coded as a dichotomous variable which takes on the value “1” if the respondent has selected the answering option “Receive help” and was set to “0” otherwise. This variable was computed for all respondents (n = 4,496).

The second dependent variable, *unmet need*, was created for the subsample of those who either reported having needed, asked for, or received support (n = 827). It was coded as a dichotomous variable taking on the value “0” if respondent had selected the answering option “Receive help” and taking on the value “1” if the respondent had selected the answering options “Need help” or “Ask for help” but not “Receive help”. It was set to missing if the respondent had not selected any of the five answering options or only the options “Offer help” or “Help someone”.

*Type of support* was only asked to those who reported receiving support. These respondents were presented five different types of support, from which they should choose all that applied. Each type was captured as a dichotomous variables (1 = yes). “Someone has helped me with grocery shopping or running errands” was chosen by almost three out of four help receivers (73%) and will in the following be referred to as *practical help.* “Someone has looked after my children” was chosen by 14% of support receivers and will hereafter be referred to as *support with childcare.* “Someone has helped me with money” was chosen by 13% of support receivers and will hereafter be referred to as *financial aid.* “Someone has supported me emotionally” was chosen by 46% of support receivers and will hereafter be referred to as *emotional support.* ”Something else, namely…” was followed by an open text field in which respondents could specify. This category was selected by 17% of support receivers and will hereafter be referred to as *Other type of support.* Receiving multiple types of support was reported quite often (47% of support receivers).

Based on these answers, we created a *categorical variable with a nominal scale level* depicting the (most) specific type of support received. There was a large overlap in receiving several types of support. Practical support was most frequently named (73% of all receivers) but is also the least specific type. We thus coded the less frequent and more specific types of support as distinct categories. Respondents were coded as receiving “childcare” if they selected that dummy variable, even if they also selected another type of support (15% of all receivers). Thereafter, the remaining respondents were selected as providing “financial aid” if they selected the respective category (plus eventually emotional, practical, or other support) (10% of all receivers). Thereafter, the remaining respondents were selected as providing “emotional support” if they selected that option (plus eventually also practical or other support) (34% of all receivers). Finally, we coded those respondents as receiving “practical support” if they had only selected the respective category (plus eventually “other”), but not childcare, financial, or emotional support (46%). This group is used as the base category in the following models. Those who had only selected “other” forms of support (n = 26)^4^, as well as all respondents who had not received support were set to missing. All in all, we have valid information on the type of support received for 627 individuals.

### Independent Variables

*“Classic” life course risks* are measured with age, health, parenthood, and employment situation. *Age* was grouped into four categories (18 to 34 years, 35 to 49 years, 50 to 64 years, and 65 years or older), which depict typical age-related life course stages. *Health* was measured using a self-reported variable with five categories ranging from “Severe illness” to “Very good health”. It was included into the models as a continuous variable after testing for non-linearity. *Parenthood* is measured with three categories: Those who have no children, those who have minor children in the household (including non-biological children), and those who have children outside the household. *Employment status* is measured with five categories: “Full-time employed”, “Part-time employed”, “Retired”, “Unemployed” and “Economically inactive”. The latter also include those in education, homemaking or permanently sick. *Pandemic-specific risk* is measured with a self-reported dummy variable, which indicates whether the respondent belongs to a risk group due to age or a chronic illness.

*Networks* were measured with four items, representing both strong and weak ties. Regarding strong ties, we first asked whether someone was married or lived together with a partner (in unmarried cohabitation), and created a dummy variable *partnered* which takes on the value “1” if either of these conditions apply. Second, the original data set asked for the estimated frequency of in-person meetings with family members and friends as well as private meetings with colleagues before the pandemic (using a six-point scale ranging from “Daily” to “Less than monthly” plus “Don’t know”). After testing for linearity (an assumption that we had to reject), this variable was recoded into the variable *frequency of meeting family/friends* with three categories: “At most monthly” (including the category “Don’t know”), “At most weekly”, and “Several times per week or daily”.

With respect to weak ties, we asked our respondents about their *membership in one or several association(s)* (such as sports clubs, political parties, voluntary welfare associations or a parents’ board at the local school). It was measured using three answering categories: “None”, “One”, “Two or more”. Fourth, we asked for the typical *frequency of attending religious service* in one’s religious community (irrespective of the denomination, thus formulated neutrally). We used a five point scale ranging from “Once per week or more often” to “Never”, plus a sixth category “Don’t know” which was set to missing. The variable was recoded with higher values representing higher frequencies and is included into our models as a continuous variable after testing for non-linear effects. Unfortunately, no further information on informal weak ties (e.g., direct contacts in the neighbourhood, or private contacts with colleagues) is available in our data. Since we can control for having minor children and employment status, we at least have a rough proxy for these network potentials.

Finally, following the original model by Andersen and Newman (1973), we consider predisposing factors as control variables. They include both attitudes and socio-demographic variables. Attitudes are represented using the standard measure for *generalized trust*. Trust enables social interactions and promotes relying on network ties (Uslaner, 2002). Moreover, trust enabled the emergence of helping arrangements (Bertogg & Koos, 2021), and was an important factor for well-being during the pandemic (McNamara et al., 2021; Shanahan et al., 2020). This variable was approximately normally distributed. After testing for non-linearity, we keep the original continuous scale, which is widely used in many large-scale surveys, such as the European Social Survey.

*Socio-demographic characteristics* are measured with six variables. Gender was measured with a dichotomous variable (0 = male, 1 = female). The highest educational level attained was measured as a categorical variable with four groups: “At most compulsory schooling”, “Upper secondary-level certificate”, “A-levels”, “Higher education certificate”. Income, on the other hand, may make personal ties redundant, as services can be bought (e.g., food delivery). Monthly net household income was collected on a continuous scale. For those respondents who refused an exact answer on their income, we presented five categories: < 900 Euros, 900–1499 Euros, 1500–4000 Euros, 4000–5999 Euros, and more than 6000 Euros. The continuous income variable was recoded into the same categories. We further included a measure of migration background (1 = yes) if the respondent or both his/her parents had been born outside of Germany. Respondent’s place of residence distinguished between urban (1 = “Large city”/“Suburban area”) or rather rural (0 = “Small town”/“Village”), and whether it is located on territory of the former German Democratic Republic (GDR) (1 = yes). Finally, we control for the process-generated variable which records the total answering time of the survey, measured in seconds, as well as for its square term.

### Analytical Strategy

For those dependent variables which are measured as dichotomous outcomes (receiving, unmet need), we apply logistic regression models. For the dependent variable which occupies a nominal scale level (type of support), we apply multinomial logistic regression models. Both model types come with the challenge that unobserved heterogeneity likely affects the effect sizes of the coefficients (Mood, 2010). Hence, logit coefficients or Odds Ratios (or Relative Risk ratios) are not comparable across different models and samples. We thus present all estimates as Average Marginal Effects (AME), which can be interpreted in terms of percentage points likelihood. Moreover, since in multinomial logistic regressions, coefficients are not intuitive to interpret, we further display the effects of our main variables graphically for these models.

Stepwise modelling with separate inclusion of relevant groups of factors was conducted: First, risk factors, then, sociodemographic characteristics and trust as controls, and finally networks. The rationale behind this order of inclusion is that networks are highly contingent on social class and gender, which is why we should interpret these effects only net of these characteristics. We also conducted a number of robustness checks, with alternative operationalizations of dependent and independent variables. The latter are consistent with our findings, unless otherwise discussed in the results section.

## Results

### Descriptive Findings

**Fig. 2:**
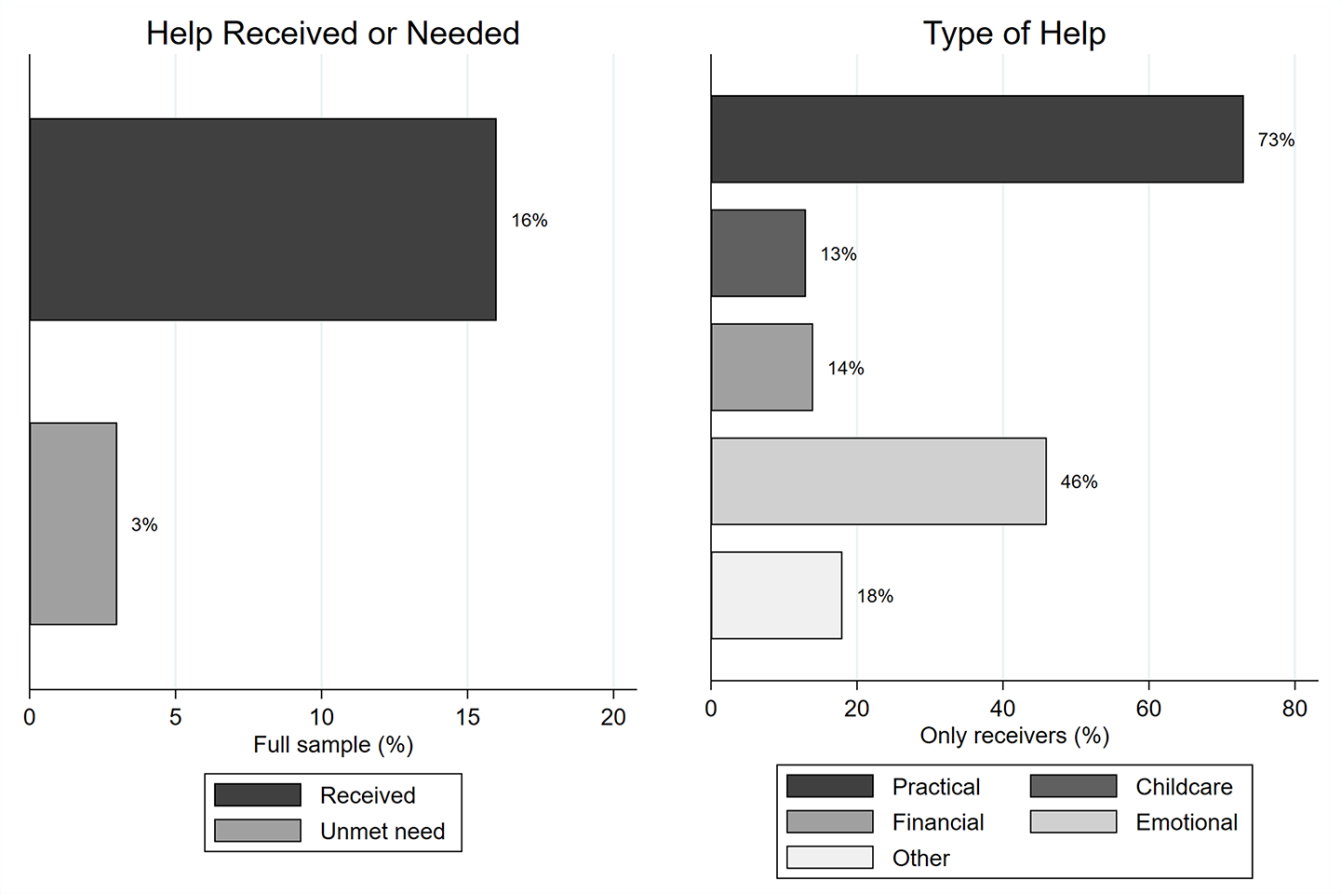
Percentage of support receivers and their type of support received. Legend: Data source (blinded). Own calculations, respondents aged 18–98 years in Germany. Percentages of whole sample (Received; unmet need—dark grey bars) respectively of receivers (Type of support—light grey bars). Multiple responses were allowed; the percentages of the different types of support thus add up to over 100%

Around one in six respondents (16%) reported receiving some form of support during the first wave COVID-19 pandemic (Fig. 2, left side). Only a small percentage (n = 144 or 3%) have unmet need. Of those respondents who received support (the right side), about three in four reported receiving practical support (73%), and about half (46%) reported receiving emotional support. Childcare and financial transfers were received about equally often, namely by about one in seven respondents (13% respectively 14%). Almost one in five respondents chose the “other” category. The different types of support add up to more than 100 per cent since multiple mentions were possible.

### Multivariate Findings

*Support Receipt and Unmet Need*

Let us now turn to the question how life course and pandemic-specific risks are associated with receipt of support (Table 1, first column). As expected (H1a), both the oldest and the youngest age groups are more likely to receive support than the other two age groups. This is indicated by the finding that the oldest age group is not significantly different from the youngest age group (the reference) in terms of receipt of support, whereas the middle two age groups are significantly less likely to receive support than the reference group. Having minor children is also associated with a higher likelihood of receiving support (H1b). Moreover, in line with our hypothesis, the better one’s the lower the likelihood of receiving support (H1c). With regard to pandemic-specific risks, we assumed that being in a risk group increases the receipt of social support (H3a), which can be confirmed by our analysis.

Next, we turn to the role of networks in the receipt of support. Being partnered is associated with less support received, which is in line with our hypothesis (H4a). We also find that individuals who had met more frequently with strong ties (family members and friends) before the pandemic are more likely to receive support, confirming H4c. Regarding weak ties, we find that the more frequently one attended services of their religious community, the more likely one is to receive support, which is in line with H5a. However, those who are a member of one or several associations are not more likely to receive support than those who are not, which contradicts this assumption. An interpretation could be that the closures of all non-essential facilities has also affected associations, diminishing the support potential of these otherwise important weak ties. An alternative explanation could be that most individuals were able to rely on others in their informal network and did not need to reach out for support via formal networks.^5^

In the next step (see the second column in Table 1), we investigate whether respondents report unmet need (1 = yes) or receive support (the reference category). For most “classic” and pandemic-specific risks, we find no difference in unmet need between the respective categories. An exception is the oldest age group (65 years or older), who are less likely to report not having received support despite having a higher likelihood of needing it. This age group is well protected against unmet need. One explanation for this finding could be that during the first phase of the pandemic in Germany, public and policy discourses had strongly centred on protecting this age group. Such a discourse may have generated a high awareness for the support needs among this group and may consequently have motivated (sufficient) support offers.

**Table 1:** AME for Receiving Support and Unmet Need

Dependent variable	Receive (Ref.: Not receive)	Unmet need (Ref.: Receive)
Sample	A	B
*Life course & pandemic-specific risks*		
Age in groups: 18–34 years (ref.)		
35–50 years	− 0.060^**^	− 0.025
51–64 years	− 0.106^***^	0.018
65 years or older	− 0.013	− 0.102^*^
Self-reported health	− 0.044^***^	0.017
Belongs to risk group	0.053^***^	0.036
No children (ref.)		
Minor in household	0.106^***^	− 0.036
Outside the household	0.023	− 0.073^*^
Full-time employed (ref.)		
Part-time employed	0.005	0.009
Retired	0.047^*^	− 0.054
Unemployed	− 0.008	− 0.044
Economically inactive	− 0.007	0.018
*Networks*		
Partnered	− 0.053^***^	0.073^*^
Meeting family/friends: At most monthly (ref.)		
Monthly to at least weekly	0.011	− 0.080^*^
Several times per week or daily	0.036^**^	− 0.090^*^
Membership in association(s): None (ref.)		
One	0.007	− 0.070^*^
Two or more	0.015	− 0.022
Frequency of attending religious service	0.040^***^	0.004
*Control variables*		
Generalized trust	0.004^*^	− 0.001
Gender: female	0.051^***^	− 0.015
Highest Education: Max. compulsory (ref.)		
Max. Secondary Level	− 0.017	− 0.043
Max. A-Levels	0.022	− 0.075
Tertiary	− 0.008	− 0.055
Monthly household income: < 900 EUR	0.030	0.024
900–1499 EUR (ref.)		
1500–3999 EUR	− 0.000	− 0.013
4000–6000 EUR	0.008	− 0.061
> 6000 EUR	0.029	− 0.032
Migration background	0.032	− 0.025
Urban area	0.005	0.060^*^
Former GDR	0.005	− 0.008
Interview time (in seconds)	0.000^***^	− 0.000^**^
Interview time (in seconds), squared	− 0.000^**^	0.000^**^
*N*	4496	826
*Pseudo-R-Squared*	0.10	0.10

Legend: Data source (blinded). Sample A: Respondents aged 18–98 years in Germany. Sample B: Respondents aged 18–98 years in Germany, who have self-reported needing or receiving support. Own calculations, AME from logistic regression model. ^*^ *p* < 0.05, ^**^ *p* < 0.01, ^***^ *p* < 0.001

With regard to the role of networks in explaining unmet need, those who had met their family members and friends more than once a month are less likely to report unmet need, supporting H4d. Similarly, those who are a member in one (but not several) association(s) are less likely to report unmet need, supporting H5b. The frequency of attending religious services is not significantly associated with unmet need, however. Finally, being partnered increases the likelihood of unmet need. This finding is surprising and contradicts our expectations (H4b). Yet, one’s partners could be part of a risk group, so that one cannot leave the house since this increases the likelihood of bringing the disease back home. Unfortunately, we cannot control for this information.

*Type of Support*.

**Fig. 3:**
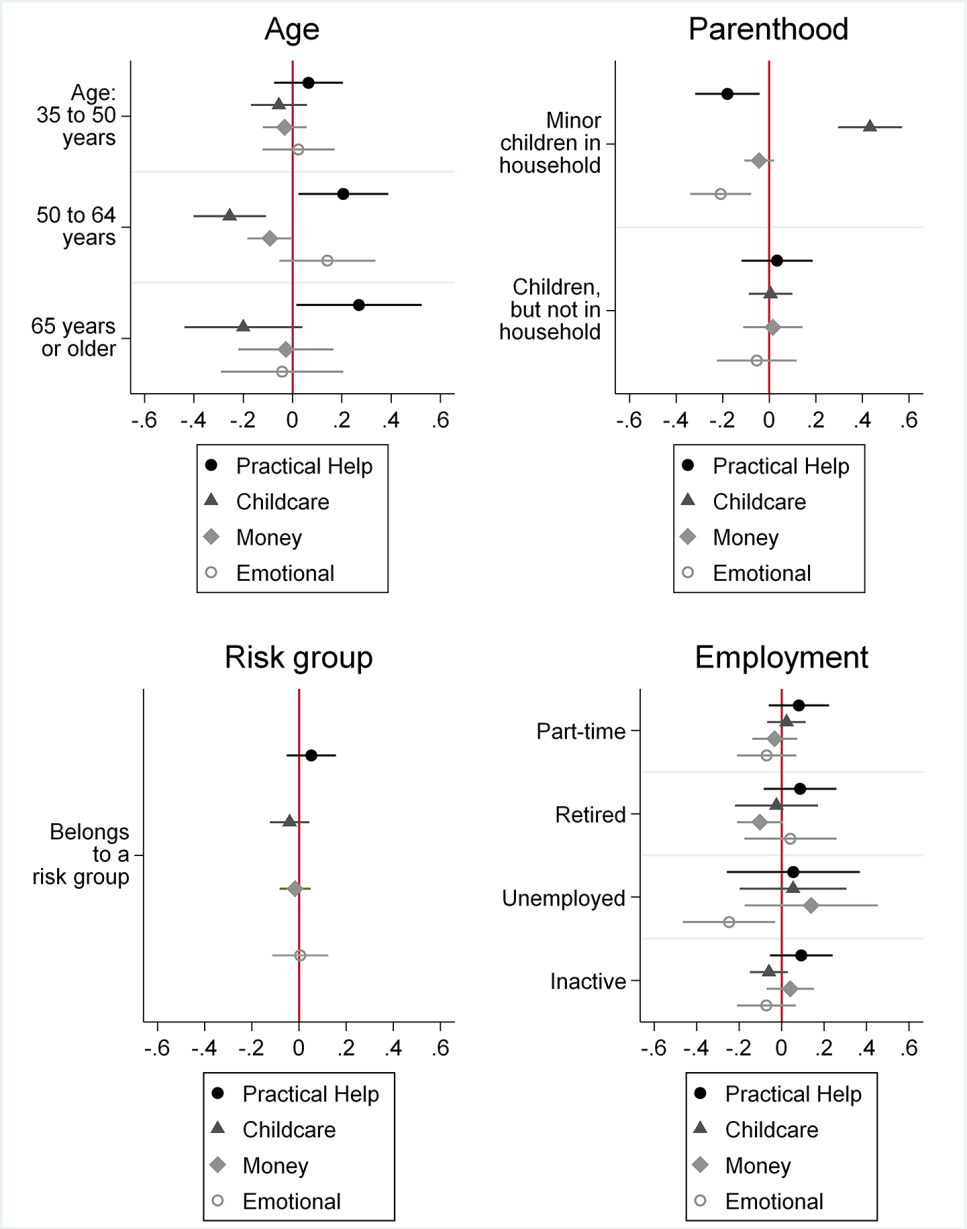
Type of Support Received by Life Course Risks (Logit Coefficients). Legend: (blinded), respondents aged 18–98 years in Germany. Own calculations. Multinomial logistic regression models including all controls from Table 1. Coefficients: Increase in likelihood of receiving a specific type of social support in comparison with reference group. Reference groups: 18–34 years, Not risk group

Finally, we address the question how different types of support depend on life course and pandemic-specific risks, or networks. This analysis is conducted on the subsample of those who report receiving support and indicating at least one specific type (n = 627). We apply a multinomial logistic regression model with “practical support” as the base category. Table 2 presents the AME from the full model, Fig. 3 presents the coefficients for selected explanatory variables.

Older age increases the likelihood of receiving practical support as compared to the young adult (top left panel in Fig. 3), which is in line with our expectation (H2b). The older a respondent, the less likely they are to receive support with childcare (supporting H2a). With regard to belonging to a risk group (bottom left panel in Fig. 3), we find no significant differences in receiving any of the four types of support. This contradicts our expectation (H3b). The same also applies to self-reported health (H2c, not depicted). Additional analyses have shown that both variables are significant in a bivariate model but become insignificant when including age and the respective other variable (risk group / health).

With regard to parenthood (top right panel of Fig. 3), we find that parents of minor children are more likely to receive childcare, and less likely to receive practical or emotional support. This is in line with our expectations (H2a). Having children but not in the household (presumably adult children) does not explain the type of support received. Finally, we turn to employment status (bottom right panel of Fig. 3). We find that those who are unemployed are less likely to receive emotional support. This speaks for workplace contacts as one important of weak ties, which may be crucial for well-being.

**Table 2:** AME for Different Types of Support (only Receivers)

Type of Support	Practical	Childcare	Money	Emotional
Sample	C	C	C	C
*Life course and pandemic-specific risks*				
Age in groups: 18–34 years (ref.)				
35–49 years	0.075	− 0.076	− 0.074	0.075
50–64 years	0.212^**^	− 0.240^***^	− 0.105^*^	0.133
65 years or older	0.267^**^	− 0.221^**^	− 0.096	0.050
Self-reported health	0.002	− 0.000	− 0.004	0.002
No children (ref.)				
Minor in household	− 0.165^**^	0.363^***^	− 0.048	− 0.149^*^
Outside the household	0.027	0.052	0.012	− 0.090
Full-time employed (ref.)				
Part-time employed	0.173^**^	0.002	− 0.047	− 0.127^*^
Retired	0.158^*^	− 0.071	− 0.084	− 0.003
Unemployed	0.276^*^	− 0.068	0.034	− 0.242^*^
Economically inactive	0.142^*^	− 0.068^*^	0.027	− 0.102
Belongs to risk group	0.058	− 0.035	− 0.029	0.006
*Networks*				
Partnered	0.023	0.024	− 0.023	− 0.023
Frequency of attending religious service	− 0.053	0.021	0.029	0.003
Frequency of meeting family/friends: At most monthly (ref.)				
More than monthly to at least weekly	− 0.048	− 0.059	− 0.019	0.127^**^
Several times per week or daily	− 0.075	− 0.065^*^	− 0.012	0.152^***^
Membership in association(s): None (ref.)				
One	− 0.032	0.011	− 0.037	0.057
Two or more	0.024	− 0.021	− 0.018	0.014
*Control variables included*	yes			
N	627			
Pseudo-R-Squared	0.29			

Legend: Data source (blinded). Sample C: Respondents aged 18–98 years in Germany who provided support and indicated which type of support (not “other”). AME from the multinomial logistic regression model. ^*^ *p* < 0.05, ^**^ *p* < 0.01, ^***^ *p* < 0.001

Finally, informal networks also affect the type of support received (Table 2). Those who met with friends or family members at least monthly before the pandemic are more likely to report receiving emotional support. This finding is feasible, as strong ties are an important resource for psychological support and emotional support is a communicative activity (Ermer & Proulx, 2019).

## Discussion and conclusion

The COVID-19 pandemic and ensuing lockdown measures have far-reaching consequences for the lives of many people, amplifying existing and creating new need for social support. Existing risks necessitate different types of social support across the life course. Yet, the pandemic has also created new needs, which are partly independent of classic life course risks. In this paper, we ask who receives support and who is left behind despite facing need; moreover, we analyse which type of support is received.

To understand patterns of receiving informal social support during the pandemic, we extend the classic model by Andersen and Newman (1973) with a life course and social network perspective and introduce the concept of pandemic-specific risks. We developed specific hypotheses pertaining to the receipt of support, unmet need and the type of support received. We argued that while risk factors (both life course and pandemic-specific) play an important role in structuring need, it is the social networks, which channel and coordinate demand and supply of need. This should become especially evident, when analysing who reports unmet need. The two risk factors should additionally allow to understand the type of support.

Our results show that one in six respondents received some type of support, and only a small minority, namely three per cent of our sample, was left with unmet need. Beside practical support, emotional support is the second most frequent type. As presumed from the theoretical model, life-course and pandemic-specific risk factors shape the need for support, but do not account for unmet need. An exception is the finding that those aged 65 years are better protected against unmet need than other age groups. Public debates about the need to protect older citizens may have generated awareness about support needs of this group and motivated support offers.

Moreover, different life course stages are linked to specific types of support. Age and parenthood seem to be the prime factors structuring different types of support. This is in line with previous literature (Broese van Groenou & De Boer, 2016). However, the receipt of support also strongly depends on the availability of informal and formal network ties (Antonucci et al., 2010). More specifically, we found that unmet need is especially prevalent among those with smaller social networks, both in terms of strong ties, and weak, formal network connections through associational membership. Yet, by combining the life course perspective with a social network perspective, and by adding the new concept of pandemic-specific risk, we move beyond studies that mainly focus on networks (Beggs et al., 1996; Gauthier et al., 2021) as well as studies that mainly focus on risks (Kasapoglu et al., 2004) and show how both matter to explain social support. In addition, by analysing unmet need, we study an important but mostly overlocked aspect to the study of social support during crisis (Kaniasty et al., 1990).

Thus, this study adds to the academic literature on the consequences of the COVID-19 pandemic, the sociological literature on life course and social determinants of social support more generally, and the literature of the role of support during societal crises and disasters (Kutak, 1938; Kaniasty et al., 1990; Kasapoglu et al., 2004). We contribute to existing research both theoretically and empirically. Our conceptual model—which takes the perspective of receivers, rather than providers of support—suggests specific social mechanisms explaining social support, which are largely supported by our empirical analyses. Thus, the model might be a fruitful theoretical framework for other scholars, who study social support or social cohesion during societal crises. Moreover, our empirical analysis provide insight into social support during a unique historical situation in Western Europe, the first lockdown during the COVID-19 pandemic. In a situation of high uncertainty about the health and economic implications of the evolving crisis, civic solidarity has complemented, and partly substituted, formal social support arrangements, leaving only a small group without sufficient support.

The study also contributes to several policy objectives. In an unfolding crisis, understanding who receives support and who does not despite need is crucial. By taking the perspective of the receivers and by additionally examining unmet need, we are able to identify marginalized groups who might become the “losers” of the pandemic (Perry et al., 2021; Gauthier et al., 2021). Identifying those who are left out is useful for developing more inclusive social policies for times of crises, which effectively protect against “new social risks” (Taylor-Gooby, 2004). Second, it is crucial to understand which role the integration into social networks play for receiving support, respectively whether and when networks can protect against unmet need. In sum, our results contribute a better understanding of when and how civil society ensures the well-being of people in need during a crisis, but also where the limits of such support are. This is relevant, as a number of studies on the role of social support for well-being during the pandemic indicate (Ohlbrecht & Jellen, 2020; Vagni, 2021).

Nevertheless, our analyses have some limitations which future research needs to address. First, using an online survey might introduce bias, e.g., if groups without sufficient internet access are under-represented. For instance, unmet need might be higher among people without internet access or digital skills. Second, we have no information on the frequency with which support was received. Third, more detailed measures of informal and formal networks, such as information on the geographical proximity to one’s family members or the number of colleagues with whom one has private contact, might have improved the predictive power of our models, and allowed us to tap into further explanatory paths. Finally, future research should investigate the long-term stability of these newly established helping relations and address the question whether lacking support adds “scars” to affected individuals life courses and well-being or might reinforce social inequalities.

## Footnotes

1 See Bradley et al., 2002; Travers et al., 2020.

2 The survey contained four modules. One basic module focused on socio-demographic factors. One module addressed questions of pandemic governance. A third module, on which the current study is based, focused on issues of solidarity. A final module addressed pandemic labour market risks. The authors of this study were responsible for designing the module on solidarity.

3 In German—other than in English—the term “help” (Hilfe) is synonymous to the term “support” and comprises a broad spectrum of different support forms. Moreover, “Hilfe” is much more common in everyday language than the term “support” (Unterstützung). In order to activate respondents‘ memories of having received support, we used the term more common in everyday language.

4 The types of support mentioned in the open text field were heterogenous and not easily attributable to a distinct support type.

5 Estimating interactions between strong (informal frequent meetings with friends) and weak ties (associational membership and church attendance) in order to examine whether strong and weak ties complement or reinforce each other. These, however, did not turn out a statistically significant result, indicating that the usage of weak ties is not influenced by the strength of strong ties.
